# Nicotinamide phosphoribosyltransferase expression and clinical outcome of resected stage I/II pancreatic ductal adenocarcinoma

**DOI:** 10.1371/journal.pone.0213576

**Published:** 2019-03-11

**Authors:** Katelynn Davis, Craig D. Dunseth, Sarah L. Mott, Kimberly L. Cramer-Morales, Ann M. Miller, Po Hien Ear, James J. Mezhir, Andrew M. Bellizzi, Carlos H. F. Chan

**Affiliations:** 1 Department of Surgery, University of Iowa, Iowa City, IA, United States of America; 2 Department of Pathology, University of Iowa, Iowa City, IA, United States of America; 3 Holden Comprehensive Cancer Center, Iowa City, IA, United States of America; 4 Department of Biochemistry, University of Iowa, Iowa City, IA, United States of America; Wayne State University, UNITED STATES

## Abstract

**Background:**

Nicotinamide phosphoribosyltransferase (NAMPT) plays a key role in the biosynthesis of nicotinamide adenine dinucleotide (NAD^+^), which is a vital cofactor in redox reactions and a substrate for NAD^+^ consuming enzymes including CD38, PARPs and sirtuins. NAMPT over-expression has been shown in various cancers and its inhibition decreases cancer cell growth, making it an attractive therapeutic target. Here we examine the NAMPT expression in a large cohort of resected stage I/II pancreatic ductal adenocarcinomas (PDAs) and correlate its expression with clinical outcomes and pathologic features.

**Methods:**

A retrospective review of patients with PDAs was conducted at a single institution. Tissue microarrays (TMAs) containing primary PDAs and their metastatic lymph nodes (mLNs) were constructed and stained for NAMPT expression. Each TMA core was evaluated for staining intensity of cancer cells (0 = no staining, 1+ = weak, 2+ = moderate, 3+ = strong) and a mean score was calculated for each case with at least two evaluable cores. NAMPT expression was correlated with clinicopathological variables using chi-squared or Fisher’s exact test, and *t*-tests for categorical and continuous variables, respectively. Survival probabilities were estimated and plotted using the Kaplan-Meier method. Cox proportional hazards regression was used to assess the effects of NAMPT staining values on recurrence-free survival (RFS) and overall survival (OS). This study was conducted under an approved IRB protocol.

**Results:**

173 primary PDAs had at least 2 TMA cores with identifiable cancer cells. The mean IHC score was 0.55 (range: 0 to 2.33). The mean IHC score of mLNs was 0.39 (range: 0–2), which was not significantly different from their primary tumors (mean IHC score = 0.47, P = 0.38). Sixty-four percent (111/173) of PDAs were positive for NAMPT staining. Stage II tumors were more likely to be positive (68% of 151 vs 41% of 22; P = 0.01). Non-obese non-diabetic patients were more likely to have NAMPT+ tumors (43.7% vs. 27.9%, P = 0.04). While RFS and OS were not statistically different between NAMPT+ vs. NAMPT- PDAs, patients with NAMPT- tumors tended to have a longer median OS (26.0 vs. 20.4 months, P = 0.34).

**Conclusion:**

NAMPT expression was detected in 64% of stage I/II PDAs and up to 72% in non-obese non-diabetic patients. Frequency of NAMPT expression correlated with pathological stage, consistent with published literature regarding its role in cancer progression. While RFS and OS were not statistically significantly different, patients with NAMPT+ PDAs tended to have a shorter survival. Thus, NAMPT inhibition may prove beneficial in clinical trials.

## Introduction

Pancreatic ductal adenocarcinoma (PDA) is one of the deadliest cancers in the United States, with a 5-year overall survival rate of 7% [1]. While surgery is the only curative treatment, most patients are not surgical candidates due to late presentation and have cancer recurrence soon after surgery; hence, response to systemic chemotherapy dictates their overall survival. However, response rate remains poor with current standard systemic chemotherapy [2].

Nicotinamide adenine dinucleotide (NAD^+^) is a vital cofactor in redox reactions and is also a substrate for many NAD^+^ consuming enzymes, including sirtuins, poly(ADP-ribose) polymerases (PARPs) and CD38 [3, 4]. Nicotinamide phosphoribosyl transferase (NAMPT), the rate-limiting enzyme in the NAD^+^ salvage pathway, converts nicotinamide to nicotinamide mononucleotide, which is a direct precursor to NAD^+^. NAMPT has been shown to be over-expressed in various cancer types including melanoma and cancers of the breast, colon, esophagus, stomach, pancreas, ovary, prostate, and thyroid [5–14]. Inhibition of NAMPT by specific inhibitors, such as FK866, or its down-regulation by siRNA reduces intracellular NAD^+^ levels and decreases cancer cell growth [5, 7, 10, 11, 15]. Inhibition of NAMPT has also been shown to increase susceptibility to cellular oxidative stress and potentiate chemotherapeutic effect [9, 13, 16, 17]. Thus, NAMPT is an attractive metabolic target for cancer treatment. Currently, one active clinical trial is being conducted using a NAMPT inhibitor in solid tumors (ClinicalTrials.gov Identifier: NCT02702492).

While NAMPT can be an attractive target, limited data currently exists regarding the expression levels of NAMPT in tumor samples derived from patients with PDAs. Barraud et al. showed variable NAMPT expression by quantitative RT-PCR and variable sensitivity to FK866 in 23 patient-derived PDA cell lines [11]. In this study, we aim to determine the expression of NAMPT in a large cohort of resected stage I/II PDAs using tissue-microarrays (TMAs) constructed at the University of Iowa and to correlate tumor NAMPT expression with overall survival (OS) and recurrence-free survival (RFS) of patients with PDAs.

## Materials and methods

### Cell lines

BxPC3, MiaPaCa2, and Panc1 human pancreatic ductal adenocarcinoma cells were purchased from ATCC (Manassas, VA) and cultured using the conditions recommended by ATCC.

### Patient population

This study was approved by the University of Iowa Institutional Review Board. A retrospective review was performed using the Institutional Oncology Registry and Electronic Medical Records of patients who were diagnosed with PDAs between 1996 and 2014. Patients were selected based on the availability of tissue from the primary tumor from the surgical pathology archives of the University of Iowa Hospitals and Clinics. Only patients with pathologically proven resected PDAs with available clinical data and tumor tissue blocks on the PDA tissue microarray were included in this study. Patients were excluded with the following criteria: no resection (biopsy tissue only), 90-day post-operative mortality, and fewer than 2 TMA tissue cores containing identifiable pancreatic cancer cells.

### PDA tissue microarrays

All pathology slides and tissue blocks were reviewed by AMB. From the pathology archive, 185 patients with pathological confirmation of PDAs were selected. Seventy of these patients also had tissue blocks with metastatic lymph nodes (mLNs). Tissue microarrays were constructed using the Manual Tissue Arrayer MTA-1 (Beecher Instruments, Sun Prairie, WI), with the 255 tumors (185 primary tumors and 70 mLNs) arrayed as triplicate 1-mm cores.

### Immunohistochemistry

**Fig 1:**
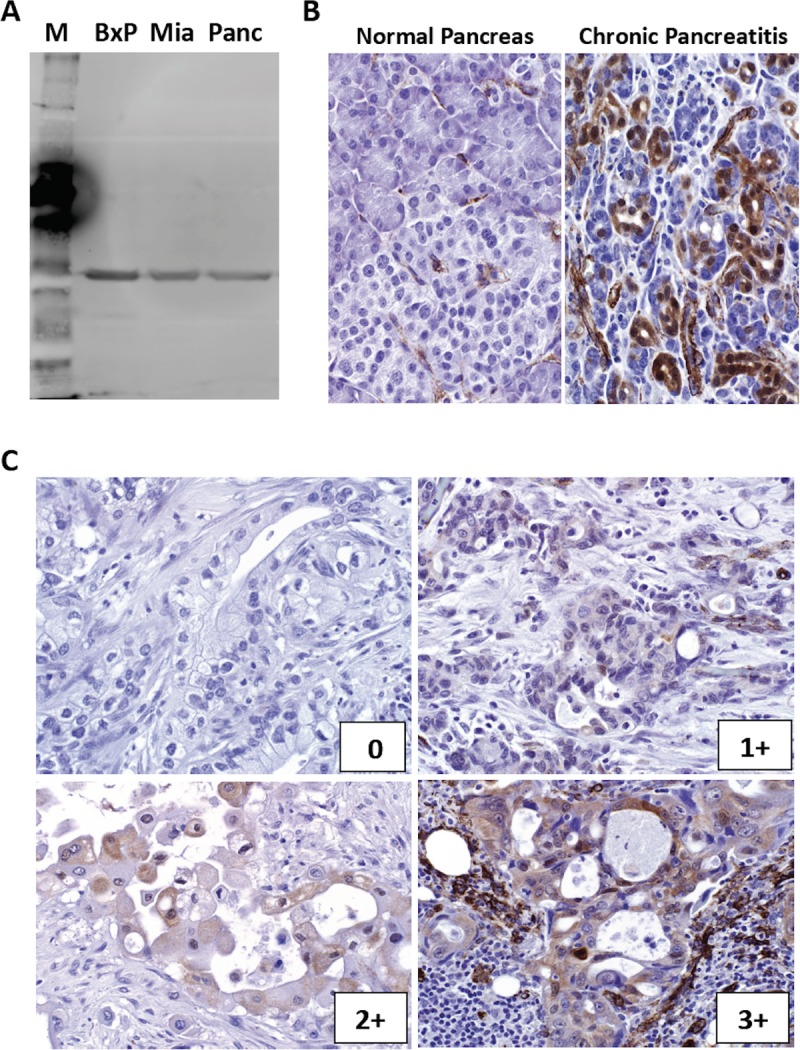
NAMPT expression in PDA. **(A)** Immunoblot analysis for NAMPT in human pancreatic cancer cell lines (BxP: BxPC3, Mia: MiaPaCa2, Pan: Panc1). Note the detection of a single band for each cell line. (**B)** Immunohistochemical staining of NAMPT in normal pancreas (left panel) and chronic pancreatitis (right panel). Note NAMPT detection (brown) in areas of chronic pancreatitis. (**C)** Immunohistochemical staining of NAMPT with difference scores: 0 (top left panel), 1+ (top right panel), 2+ (bottom left panel), 3+ (bottom right panel).

Immunohistochemical staining of NAMPT was performed using the Dako Autostainer Link 48 on 4-µm-thick tissue sections after deparaffinization, rehydration, and pressure cooker heat-induced epitope retrieval in Target Retrieval Solution (Dako, Carpinteria, CA) using anti-NAMPT mouse monoclonal antibodies (OMNI379) in 1:250 dilution for 15 minutes and Envision+ detection reagents (Dako, Carpinteria, CA) for 15 minutes. All TMA slides were scored blindly by AMB. Cancer cells were evaluated for staining intensity and given a score: 0 = no staining, 1+ = weak, 2+ = moderate, 3+ = strong (Fig 1C). The mean scores were calculated for each case. Staining in non-cancer cells such as stromal and immune cells were not accounted for the overall IHC-scores.

### Clinical data collection

Electronic Medical Records and Oncology Registry data were reviewed. Collected data included patient age, gender, race, pre-operative body mass index (BMI), diabetes status, pre-operative CA19-9 level, date of diagnosis, treatment details (dates of operation, type of operation, and neoadjuvant/adjuvant therapy), pathological data (pathological stage, tumor size, tumor grade, status of surgical margins), follow-up data (date of last follow-up, dates of recurrence if any, date of death if deceased, status of last follow-up, and site of recurrence if any). Date of last follow-up was determined as the last visit to see a physician, nurse practitioner, or physician assistant at the University of Iowa Hospitals and Clinics or at an outside hospital or clinic where their outside medical records were obtained. Disease recurrence was defined as local or metastatic recurrence on CT scan that were not present on the pre-operative or immediate post-operative CT scan as reported by the radiologist reading the scan. When the term “suspicious for recurrence” was used, this would only be considered true recurrence if later scans were consistent with recurrence and the date of recurrence was set forth on the initial scan with suspicion.

### Statistical analysis

To investigate NAMPT status differences, chi-squared (Fisher’s exact, where appropriate), and t-tests were used. Survival probabilities were estimated and plotted using the Kaplan-Meier method. Cox proportional hazards regression was used to assess the effects of NAMPT staining values on recurrence-free and overall survival. For recurrence-free survival, time was calculated from date of surgery to recurrence or death due to any cause. Patients not experiencing a recurrence or death were censored at date of last contact. For overall survival, time was calculated from date of surgery to death due to any cause. Patients still alive were censored at date of last contact. To account for potential confounding effects, adjustment for variables with a significance level of <0.10 on univariate analysis were considered for inclusion while forcing NAMPT into a multivariate model for each outcome. Estimated effects of predictors are reported as hazard ratios (HR) along with 95% confidence intervals (CI). All statistical testing was two-sided and assessed for significance at the 5% level using SAS v9.4 (SAS Institute, Cary, NC).

### Immunoblot

Cell lysates were separated on a NuPAGE gel followed by transfer to a PVDF membrane. Membranes were blocked in 5% non-fat milk and then incubated with mouse monoclonal anti-NAMPT antibodies (OMNI379; Adipogen, San Diego, CA) overnight at 4°C. Following washing, membranes were then incubated with HRP-tagged anti-mouse antibodies (7076P2; Cell Signaling Technology, Danvers, MA) and developed using Pierce ECL2 kit (Thermo Fisher Scientific, Waltham, MA).

## Results

### Patient population and clinicopathological characteristics

Of 185 patients with PDAs included in the TMAs, 173 patients had at least 2 tissue cores remaining on the TMA slides containing identifiable cancer cells after NAMPT IHC staining. Of the remaining 173 patients, average age at the time of operation was 65 years. Sixty percent of patients were male and the majority of them were white (87.9%). Pancreaticoduodenectomy was performed in 85% of patients; 9.2% of patients underwent neoadjuvant chemotherapy; 66.5% underwent adjuvant chemotherapy; and 44.6% underwent radiation therapy. Most tumors were pathological stage II (87.3%) and T3 (76.3%); 56.1% were node positive disease; and 26.6% were margin positive (Table 1).

**Table 1:** Patients’ demographics and clinicopathological data.

Variables		N (%)	Mean (Range)
Age at Operation (years)			65 (38–85)
Gender	Male Female	103 (59.5%) 70 (40.5%)	
Race	White Other Unknown	152 (87.9%) 7 (4.1%) 14 (8.1%)	
Preoperative BMI (kg/m^2^)			27.6 (16.0–47.5)
Diabetes	Yes No	43 (24.9%) 130 (75.1%)	
Elevated CA19-9 level (≥37 U/dl)	Yes No Unknown	76 (44.0%) 44 (25.4%) 53 (30.6%)	
Time to surgery (days)			32 (0–180)
Operation	Pancreaticoduodenectomy Distal pancreatectomy	147 (85.0%) 26 (15.0%)	
Neoadjuvant chemotherapy	Yes No	16 (9.2%) 157 (90.8%)	
Adjuvant chemotherapy	Yes No Unknown	115 (66.5%) 56 (32.4%) 2 (1.2%)	
Radiation therapy	Chemoradiation Radiation alone No Unknown	75 (43.4%) 2 (1.2%) 38 (22.0%) 58 (33.5%)	
Pathological stage	1A 1B 2A 2B	5 (2.9%) 17 (9.8%) 54 (31.2%) 97 (56.1%)	
Tumor stage	T1 T2 T3	10 (5.8%) 31 (17.9%) 132 (76.3%)	
Lymph node stage	N0 N1	76 (43.9%) 97 (56.1%)	
Resection margin	R0 R1 Unknown	126 (72.8%) 46 (26.6%) 1 (0.6%)	
Tumor size (cm)			3.4 (0.8–9.5)
Tumor grade	Well differentiated Moderately differentiated Poorly differentiated Unknown	8 (4.6%) 102 (59.0%) 55 (31.8%) 8 (4.6%)	
# of lymph nodes examined			12 (0–37)
# of positive lymph nodes			2 (0–16)
Length of follow-up (months)			34.8 (1.6–194.2)
Vital status at last follow up	Alive Dead	27 (15.6%) 146 (84.4%)	
Recurrence	Local Liver Peritoneum Lung	49 (28.0%) 54 (31.2%) 20 (11.6%) 19 (11.0%)	

### NAMPT expression in PDA

Using 3 different human pancreatic cancer cell lines (BxPC3, MiaPaCa2 and Panc1), a single band of NAMPT of the correct molecular weight was obtained on immunoblot analysis, showing the specificity of the monoclonal antibody used in this study (Fig 1A). While normal pancreatic tissues were generally negative for NAMPT IHC staining, normal pancreatic ductal epithelial cells showed significant (3+) NAMPT IHC staining in the areas of chronic pancreatitis (Fig 1B). Of 469 evaluable cores of primary PDA, NAMPT IHC scores were 0 in 52.8%, 1+ in 39.4%, 2+ in 6.6%, and 3+ in 1.1%. Among all 173 patients with PDAs, the mean NAMPT IHC score was 0.55 (range: 0–2.33). Sixty-two patients (35.8%) had NAMPT IHC score of 0; 55 patients (31.8%) had NAMPT IHC score of ≥1. Of 173 patients, 52 patients also had evaluable cores of their mLNs. Mean NAMPT IHC score of mLNs was 0.39 (range: 0–2), which was not significantly different from their primary tumors (mean: 0.47, P = 0.38). The NAMPT IHC scores increased in 18 mLNs and was decreased in 22 mLNs.

When NAMPT expression was stratified as negative (IHC score = 0) and positive (IHC score >0), NAMPT+ PDAs had higher pathological stage than NAMPT- PDAs (91.9% vs. 79.0% pathological stage II, P = 0.01, Table 2). In addition, obese patients tended to have NAMPT- PDAs in comparing to non-obese patients (20/43 (46.5%) vs.35/114 (30.7%), P = 0.06). While diabetes alone did not seem to impact tumor NAMPT expression, patients with obesity and/or diabetes had a significantly higher likelihood of having NAMPT- PDAs in comparison to patients without obesity and diabetes (31/71 (43.7%) vs. 24/86 (27.9%), P = 0.04).

**Table 2:** NAMPT IHC score in PDA.

Variables		*NAMPT Expression		P-value
		Negative N = 62	Positive N = 111	
Mean age at operation (years)		64.9	65.8	0.61
Gender	Female Male	24 (38.7%) 38 (61.3%)	46 (41.4%) 65 (58.6%)	0.73
Race	White Other	55 (96.5%) 2 (3.5%)	97 (95.1%) 5 (4.9%)	1.00
Obesity (BMI ≥30 kg/m^2^)	Yes No	20 (36.4%) 35 (63.6%)	23 (22.5%) 79 (77.5%)	0.06
Diabetes	Yes No	17 (27.4%) 45 (72.6%)	26 (23.4%) 85 (76.6%)	0.56
Obesity +/- diabetes	Yes No	31 (56.4%) 24 (43.6%)	40 (39.2%) 62 (60.8%)	**0.04**
Operation	Pancreaticoduodenectomy Distal pancreatectomy	54 (87.1%) 8 (12.9%)	93 (83.8%) 18 (16.2%)	0.56
Neoadjuvant chemotherapy	Yes No	4 (6.5%) 58 (93.5%)	12 (10.8%) 99 (89.2%)	0.34
Adjuvant chemotherapy	Yes No	45 (73.8%) 16 (26.2%)	70 (63.6%) 40 (36.6%)	0.18
Radiation therapy	Yes No	29 (64.4%) 16 (35.6%)	48 (68.6%) 22 (31.4%)	0.65
Pathological stage	1 2	13 (21.0%) 49 (79.0%)	9 (8.1%) 102 (91.9%)	**0.01**
Tumor stage	T1/2 T3	19 (30.6%) 43 (69.4%)	22 (19.8%) 89 (80.2%)	0.11
Lymph node stage	N0 N1	29 (46.8%) 33 (53.2%)	47 (42.3%) 64 (57.7%)	0.57
Resection margin	R0 R1	47 (75.8%) 15 (24.2%)	79 (71.8%) 31 (28.2%)	0.57
Tumor grade	Well-moderately differentiated Poorly differentiated	42 (71.2%) 17 (28.8%)	68 (64.2%) 38 (35.8%)	0.36
Median follow-up (months)		21.1	20.2	0.29
Median overall survival (months)		26	20.4	0.34
Median recurrence-free survival (months)		13.5	12	0.34
Recurrence:				
Local	Yes	18 (29.5%)	31 (27.9%)	0.83
	No	43 (70.5%)	80 (72.1%)	
Liver	Yes	17 (27.9%)	37 (33.6%)	0.44
	No	44 (72.1%)	73 (66.4%)	
Peritoneum	Yes	6 (9.8%)	14 (12.6%)	0.59
	No	55 (90.2%)	97 (87.4%)	
Lung	Yes	5 (8.2%)	14 (12.6%)	0.38
	No	56 (91.8%)	97 (87.4%)	

* Note: NAMPT+ is defined as IHC score > 0.

### Clinical outcome

**Fig 2:**
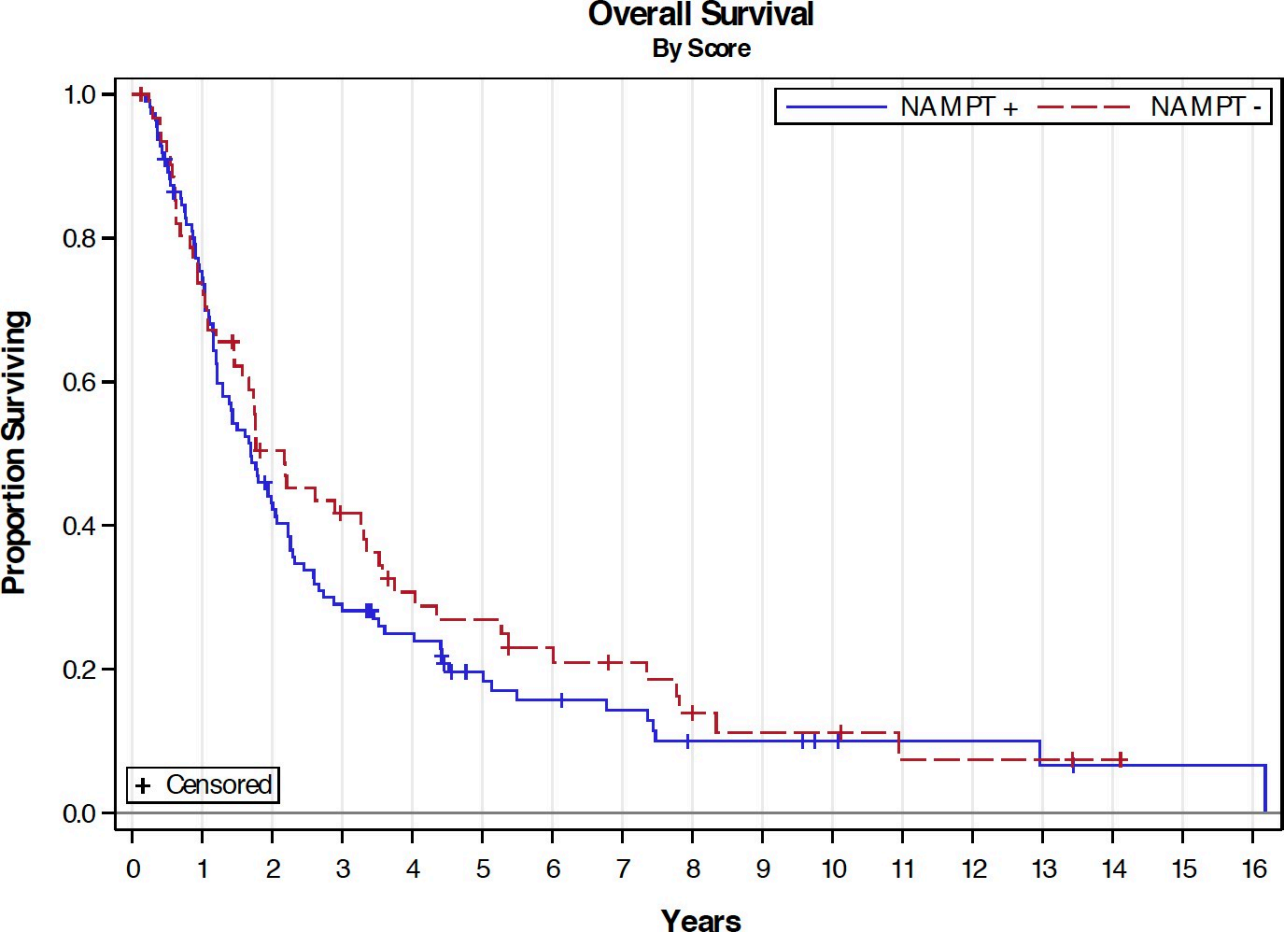
Overall survival by NAMPT IHC score. Kaplan-Meier curves showing overall survival of patients with NAMPT+ (blue line) and NAMPT- (red line) PDA. Median OS: 20.4 months (NAMPT+) vs. 26.0 months (NAMPT-); *P*-value: 0.34.

**Fig 3:**
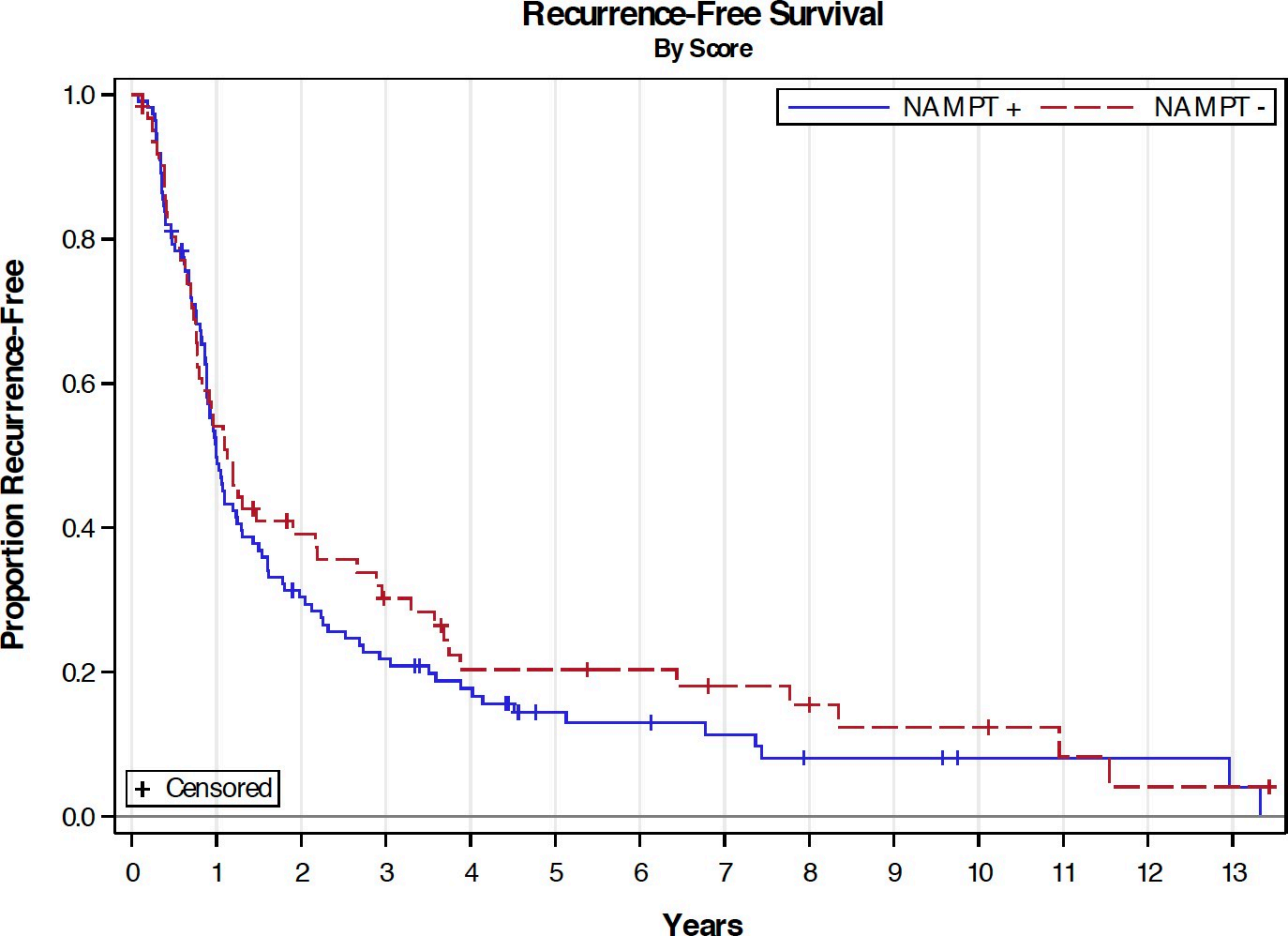
Recurrence-free survival by NAMPT IHC score. Kaplan-Meier curves showing recurrence-free survival of patients with NAMPT+ (blue line) and NAMPT- (red line) PDA. Median OS: 12.0 months (NAMPT+) vs. 13.5 months (NAMPT-); *P*-value: 0.34.

With a median follow-up of 20.9 months, patients with NAMPT+ PDAs tended to have a shorter OS than those with NAMPT- PDAs (median 20.4 months vs. 26.0 months, P = 0.34), although it was not statistically significant (Fig 2). RFS and sites of recurrence were similar between the two groups (Fig 3 and Table 2). In the univariate analysis of RFS (Table 3), pathological stage I disease, negative resection margin and adjuvant chemotherapy were significantly associated with improved RFS (P < 0.05). Pancreaticoduodenectomy tended to have worse RFS with a hazard ratio of 1.50 in comparing to distal pancreatectomy (P = 0.09). In the univariate analysis of OS (Table 4), negative resection margin and adjuvant chemotherapy were significantly associated with improved OS (P < 0.01). While not statistically significant, pancreaticoduodenectomy, pathological stage II, tumor stage T3, and positive lymph nodes tended to have worse OS with hazard ratios between 1.35 and 1.66 in comparing to distal pancreatectomy, pathological stage I, tumor stage T1/2 and negative lymph node, respectively (P-values: 0.06–0.09). In the multivariate analysis, only adjuvant chemotherapy and negative resection margin were significantly associated with improved RFS (Table 5) and OS (Table 6).

**Table 3:** Univariate analysis for recurrence-free survival.

			Recurrence-Free Survival			
Covariate	Level	N	Hazard Ratio	95% CI		P-value
Gender	Male	103	1.05	0.75	1.47	0.76
	Female	70	Ref	-	-	-
Obesity (BMI ≥30 kg/m^2^)	Yes	43	1.15	0.78	1.68	0.48
	No	114	Ref	-	-	-
Diabetes	Yes	43	1.10	0.76	1.58	0.62
	No	130	Ref	-	-	-
Obesity +/- Diabetes	Yes	71	1.17	0.83	1.65	0.36
	No	86	Ref	-	-	-
Operation	Pancreaticoduodenectomy	147	1.50	0.93	2.40	0.09
	Distal Pancreatectomy	26	Ref	-	-	-
Neoadjuvant chemotherapy	Yes	16	1.19	0.70	2.04	0.52
	No	157	Ref	-	-	-
Adjuvant chemotherapy	No	56	1.45	1.03	2.03	**0.03**
	Yes	115	Ref	-	-	-
Radiation therapy	Yes	77	1.07	0.69	1.66	0.75
	No	38	Ref	-	-	-
Pathological stage	2	151	1.73	1.04	2.88	**0.04**
	1	22	Ref	-	-	-
Tumor stage	3	132	1.38	0.94	2.02	0.10
	1/2	41	Ref	-	-	-
Lymph node stage	1	97	1.31	0.95	1.82	0.10
	0	76	Ref	-	-	-
Resection margin	Positive	46	1.87	1.31	2.67	**< .01**
	Negative	126	Ref	-	-	-
Tumor grade	Poorly Differentiated	55	1.23	0.86	1.77	0.26
	Well-Moderately Differentiated	110	Ref	-	-	-
NAMPT Score	>0	111	1.18	0.84	1.65	0.34
	0	62	Ref	-	-	-
NAMPT Score	1+	55	1.00	0.71	1.41	0.99
	<1	118	Ref	-	-	-
Age at Operation	Units = 1	173	1.00	0.98	1.01	0.74
Preoperative BMI	Units = 1	157	1.00	0.97	1.04	0.83
NAMPT Score	Units = 1	173	1.10	0.83	1.48	0.51

**Table 4:** Univariate analysis for overall survival.

			Overall Survival			
Covariate	Level	N	Hazard Ratio	95% CI		P-value
Gender	Male	103	0.99	0.71	1.38	0.95
	Female	70	Ref	-	-	-
Obesity (BMI ≥30 kg/m^2^)	Yes	43	1.18	0.80	1.73	0.40
	No	114	Ref	-	-	-
Diabetes	Yes	43	1.07	0.74	1.55	0.72
	No	130	Ref	-	-	-
Obesity +/- Diabetes	Yes	71	1.18	0.84	1.67	0.34
	No	86	Ref	-	-	-
Operation	Pancreaticoduodenectomy	147	1.51	0.93	2.46	0.09
	Distal Pancreatectomy	26	Ref	-	-	-
Neoadjuvant chemotherapy	Yes	16	1.25	0.72	2.18	0.42
	No	157	Ref	-	-	-
Adjuvant chemotherapy	No	56	1.76	1.25	2.47	**< .01**
	Yes	115	Ref	-	-	-
Radiation therapy	Yes	77	0.86	0.55	1.33	0.49
	No	38	Ref	-	-	-
Pathological stage	2	151	1.66	0.98	2.81	0.06
	1	22	Ref	-	-	-
Tumor stage	3	132	1.42	0.96	2.11	0.08
	1/2	41	Ref	-	-	-
Lymph node stage	1	97	1.35	0.97	1.89	0.07
	0	76	Ref	-	-	-
Resection margin	Positive	46	1.84	1.29	2.62	**< .01**
	Negative	126	Ref	-	-	-
Tumor grade	Poorly Differentiated	55	1.21	0.84	1.75	0.30
	Well-Moderately Differentiated	110	Ref	-	-	-
NAMPT Score	>0	111	1.18	0.84	1.66	0.34
	0	62	Ref	-	-	-
NAMPT Score	1+	55	0.93	0.65	1.32	0.67
	<1	118	Ref	-	-	-
Age at Operation	Units = 1	173	1.00	0.99	1.02	0.71
Preoperative BMI	Units = 1	157	1.00	0.97	1.03	0.90
NAMPT Score	Units = 1	173	1.04	0.78	1.39	0.79

**Table 5:** Multivariate analysis for recurrence-free survival.

			Recurrence-Free Survival			
			——————————————————			
Covariate	Level	N	Hazard Ratio	95% CI		P-value
Operation	Pancreaticoduodenectomy	146	1.27	0.71	2.11	0.36
	Distal Pancreatectomy	25	Ref	-	-	-
Adjuvant chemotherapy	No	56	1.92	1.33	2.76	**< .01**
	Yes	115	Ref	-	-	-
Pathological stage	2	150	1.75	1.00	3.06	0.05
	1	21	Ref	-	-	-
Resection margin	Positive	46	2.19	1.50	3.21	**< .01**
	Negative	125	Ref	-	-	-
NAMPT Score	>0	110	0.94	0.66	1.34	0.75
	0	61	Ref	-	-	-

**Table 6:** Multivariate analysis for overall survival.

			Overall Survival			
			——————————————————			
Covariate	Level	N	Hazard Ratio	95% CI		P-value
Operation	Pancreaticoduodenectomy	146	1.24	0.74	2.07	0.41
	Distal Pancreatectomy	25	Ref	-	-	-
Adjuvant chemotherapy	No	56	2.42	1.66	3.52	**< .01**
	Yes	115	Ref	-	-	-
Pathological stage	2	150	1.67	0.95	2.96	0.08
	1	21	Ref	-	-	-
Resection margin	Positive	46	2.26	1.54	3.33	**< .01**
	Negative	125	Ref	-	-	-
NAMPT Score	>0	110	0.92	0.65	1.32	0.66
	0	61	Ref	-	-	-

## Discussion

NAMPT is an attractive metabolic target for cancer treatment. Inhibition of NAMPT by specific inhibitors, such as FK866, or its down-regulation by siRNA reduces intracellular NAD^+^ level and decreases cancer cell growth in both *in vitro* and *in vivo* models [5, 7, 10, 11, 15]. Inhibition of NAMPT has also been shown to increase susceptibility to cellular oxidative stress and potentiate chemotherapeutic effect [9, 13, 16, 17]. However, NAMPT expression has not been well studied in human PDA with only one small study including 23 patient-derived pancreatic cancer cell lines [11]. In our current study with 173 analyzable patients with PDAs on the TMAs constructed at the University of Iowa, NAMPT was expressed in 64% of PDAs and up to 72% of PDAs in non-obese non-diabetic patients. It is unclear why obese or diabetic patients have a lower incidence of NAMPT expression in PDA. Obese individuals can have elevated circulating levels of extracellular NAMPT derived from their adipose tissues [3]. We have unpublished preliminary data showing elevated serum levels of NAMPT in obese and/or diabetic patients with pancreatic tumors. Similar observation has been demonstrated in obese patients with esophagogastric adenocarcinomas and breast cancer [8, 18]. We speculate that circulating NAMPT may exert a negative feedback on intracellular NAMPT expression, although further studies are required to examine such effect and to determine the underlying mechanism.

While there were no consistent changes of NAMPT expression between primary tumors and their corresponding mLNs in our study, NAMPT expression was associated with higher pathological stage of disease. This is consistent with previously published data on the proliferative effects of NAMPT expression in pancreatic cancer cells *in vitro* and *in vivo* [10, 11]. Although we could not reach statistical significance, probably due to a small sample size, we showed that patients with NAMPT- PDAs had longer median OS (median 26.0 vs. 20.4 months). One could argue the observed effect was due to stage migration since stage II disease was more likely to be NAMPT+. But the numbers of stage I disease in this study was relatively very small and would have limited impact on the survival curves. Thus, if we could downregulate NAMPT expression or function in patients with NAMPT+ PDAs, we could potentially delay cancer progression and prolong survival similar to those with NAMPT- PDAs. In fact, Barraud et al. showed that FK866, a NAMPT inhibitor, could decrease intracellular NAD^+^ level and cell viability of patient-derived pancreatic cancer cell lines and that their sensitivity to FK866 was directly related to their NAMPT expression levels [11]. Similar results were obtained by Espindola-Netto et al. using another NAMPT inhibitor, STF-118804 [19]. Interestingly, NAMPT inhibition alone may not always have impact on cancer cell survival despite their elevated NAMPT expression. This is due to the concomitant over-expression of nicotinic acid phosphoribosyltransferase (NAPRT), leading to the maintenance of NAD^+^ levels via the *de novo* synthesis pathway [20]. Due to the complexity of NAD^+^ metabolism, it may be worthwhile to evaluate the expression of NAPRT and other NAD^+^ metabolic enzymes in PDA for future studies.

Currently, one active clinical trial is being conducted using a NAMPT inhibitor in solid tumors (ClinicalTrials.gov Identifier: NCT02702492). It would be interesting to correlate NAMPT expression level to treatment response in patients from these clinical trials; and perhaps, NAMPT IHC score may be used as a molecular predictor of response. Since both Barraud et al. and Espindola-Netto et al. showed that NAMPT inhibition could augment the sensitivity of pancreatic cancer cells to chemotherapeutic agents [11, 19], it would be interesting to determine if addition of NAMPT inhibitors to current systemic chemotherapy regimens could improve survival of pancreatic cancer patients stratified by tumor NAMPT expression.

Limitations of this study include: 1) retrospective nature of the current study; 2) tumor tissues and clinical data from a single institution; 3) case selection bias based on tissue availability; and 4) long study period (1996–2014) that could affect the survival data since there were some changes of systemic treatment over time. However, we did not anticipate any significant impact on our findings and conclusion since our patients were well-balanced over the study period between the two groups and survival of PDA patients did not have significant changes across the study period. Nevertheless, further studies with larger cohorts of patients within a shorter study period is required to better evaluate the impact of NAMPT over-expression in OS and RFS of patients with PDAs.

In conclusion, we showed that NAMPT was expressed in the majority of PDAs and in higher stage disease, making NAMPT an intriguing metabolic target for treating PDA, probably in combination with current systemic chemotherapy regimens.
